# Retraction of transporting bone segment during Ilizarov bone transport

**DOI:** 10.1186/s12891-020-03702-7

**Published:** 2020-10-26

**Authors:** Yunhong Ma, Qudong Yin, Yongwei Wu, Zongnan Wang, Zhenzhong Sun, Sanjun Gu, Yongjun Rui, Xiaofei Han

**Affiliations:** 1 Department of Orthopaedics, Wuxi No. 9 People’s Hospital Affiliated to Soochow University, No. 999 Liangxi Road, Wuxi, 214062 Jiangsu China; 2 Department of Orthopaedics, Shuyang People’s Hospital, Suqian, 223000 Jiangsu China

**Keywords:** Callus distraction, Bone transport, Retraction of transporting bone segment, Force

## Abstract

**Background:**

Retraction of transporting bone segment (TBS) may occur when the fixator of the TBS is removed prior to full consolidation of the distracted callus, which has adverse effect on the healing of the docking site. However, there are few reports on the retraction of TBS. The purpose of this study is to analyze the causes and risk factors of the retraction of TBS.

**Methods:**

The clinical data of 37 cases with tibial bone defect treated by Ilizarov bone transport were analyzed retrospectively, in whom the TBS fixator was removed prior to full consolidation of the distracted callus and union of the docking site. Bivariate correlation was used to analyze relationship between the retraction distance of TBS and potential risk factors including age, gender, course, length of bone defect, number of operations, size of TBS, transport distance, timing and time interval of removal of TBS fixator. Risk factors with significant level were further identified using multivariate linear regression.

**Results:**

Bivariate correlation showed that the timing of removal was negatively correlated with the retraction distance, and the time interval and transport distance were positively correlated with the retraction distance(*p* < 0.05), the age, gender, course, length of bone defect, size of TBS and number of operations were not correlated with the retraction distance(*p* > 0.05). Multivariate linear regression of the 3 risk factors showed that the timing of removal and time interval were the main risk factors affecting the retraction distance (*p* < 0.05), but the transport distance was not (*p* > 0.05).

**Conclusion:**

The traction forces of TBS endured from the soft tissues and the unconsolidated distracted callus have elastic properties, which can make retraction of TBS. The timing of removal and time interval are the main risk factors of the retraction of TBS. In the case of early removal, another external fixation or quickly converted to internal fixation should be performed to avoid the adverse effect of more retraction.

## Background

Callus distraction (distraction osteogenesis) by Ilizarov’s method has been an effective technique to reconstruct large bone defects and correct discrepancy of limbs [1–3]. External distraction system was more common and earlier than intramedullary distraction system in Ilizarov bone transport. Removal of external fixator is usually performed after maturation of mineralization of the distracted callus and union of the docking site in traditional Ilizarov bone transport. However, traditional Ilizarov bone transport presented high rates of delayed union and nonunion of the docking site and pin-track infection or loosening, inconvenience for rehabilitation and nursing, and psychological disorder induced by long-term external fixation [3–6]. These complications and drawbacks have become the bottleneck restricting the development of this technology. Therefore, how to reduce complications and defects has become a subject of clinical research. Recently, some scholars [7–9] reported improved Ilizarov bone transport with less complications, that is, when the docking site was closed or difficult to heal, the external fixator was removed and then converted to internal fixation (plate or intramedullary nail). However, it usually takes 1–2 weeks or more for the pin-track to heal before implantation of a new implant. During the period between removal of external fixator and new implantation of internal fixation, retraction of transporting bone segment (TBS) may occur even if plaster cast is used. The retraction of TBS has adverse effect on the healing of the docking site. Previous literatures paid more attention to the bone union and complications in distraction osteogenesis, rarely reported the causes and influencing factors of the retraction of TBS [10–14]. Understanding retraction phenomenon of TBS is help to take appropriate measures to avoid adverse effect or complications. Therefore, the clinical data of 37 patients with removal of TBS fixator prior to full consolidation of the distracted callus and union of the docking site in our hospital were retrospectively analyzed to identify the causes and risk factors of the retraction of TBS.

## Methods

### Inclusion and exclusion criteria

Inclusion criteria: ① Patients with tibial defect treated by Ilizarov bone transport; ②the TBS fixator or total external fixator was removed prior to full consolidation of the distracted callus and union of the docking site. Exclusion criteria: Patients with incomplete radiographic data were excluded. This study was approved by the ethics committee of Wuxi no.9 People’s Hospital and Shuyang People’s Hospital, and written informed consents were obtained from all participants.

### Patients

Between January 2009 and December 2018, 37 cases were included in the study who were traumatic fractures with tibial bone defect. Before bone transport, all patients with bone defect were fixed with ring or single arm external fixator, among them, 5 cases were simultaneously shortened of the affected limb. There were twenty-three males and fourteen females, ranging in age from 15 to 71 years with an average age of 39.95 years.

### Observation indexes

Retraction distance: the retraction length examined by radiographic evaluation before and after removal of TBS fixator.

Course: the days from traumatic bone defect to bone transport.

Number of operations: the number of operations performed before bone transport.

Timing of removal: the time from the beginning of bone transport to the removal of TBS fixator.

Size of TBS: the length of the TBS.

Time interval: the days between removal of TBS fixator and follow-up radiographic examination of showing retraction of TBS.

Retraction distance and nine potential risk factors are shown in Table 1.

**Table 1:** Descriptive statistics of variables

Retraction distance (mm)	8.08 ± 6.39 (1.5–30)
Age (yrs)	39.95 ± 14.83 (15–71)
Gender(M/F)	22/15
Course (days)	40.54 ± 25.65 (7–112)
Size of TBS (mm)	91.03 ± 15.18 (6–15)
Length of defect (mm)	64.10 ± 11.55 (5–11.5)
Time interval (days)	9.22 ± 3.95 (6–25)
Timing of removal (Mon)	7.47 ± 1.94 (3.5–12)
Number of operations (Num)	1.65 ± 0.82 (1–4)
Transport distance (mm)	68.40 ± 13.41 (50–110)

### Statistical analysis

Data analysis was performed using SPSS 20.0 statistical software (IBM, New York, USA). Firstly, scatter diagram and bivariate correlation were used to analyze relationships between retraction distance and nine potential risk factors including age, gender, course, length of bone defect, number of operations, size of TBS, transport distance, timing and time interval of removal of TBS fixator. Risk factors with significant level were further identified using multivariate linear regression. *P* < 0.05 was considered significant.

## Results

Variables showing a correlation trend by scatter diagram analysis were the timing of removal, time interval and transport distance (Fig. 1). Bivariate correlation analysis showed that the timing of removal was negatively correlated with the retraction distance (r = − 0.832, *P* = 0.000), and the time interval and transport distance were positively correlated with the retraction distance(r = 0.368, *P* = 0.025, r = 0.337, *P* = 0.041 respectively). However, age, gender, course, size of TBS, bone defect length and number of operations were not correlated with the retraction distance (r = − 0.121, *P* = 0.475, r = 0.020, *P* = 0.907, r = − 0.247, *P* = 0.140, and r = 0.150, *P* = 0.377, r = 0.006, *p* = 0.974, r = 0.312, *p* = 0.060, respectively). Multivariate linear regression analysis of the 3 risk factors showed that the timing of removal and time interval were the main risk factors of retraction distance of TBS (Table 2), of which, the timing of removal had the greatest impact (t = − 10.171, *p* = 0.000), followed by the time interval (t = 3.193, *p* = 0.003), but the transport distance was not a main risk factor of retraction distance (t = − 0.717, *p* = 0.479). Regression equation: Y = 27.070–2.808X1 + 0.449X2. Scatter diagram of regression model of standardized predicted value of retraction distance seen Fig. 2. Typical cases are shown in Fig. 3 and 4.

**Fig. 1:**
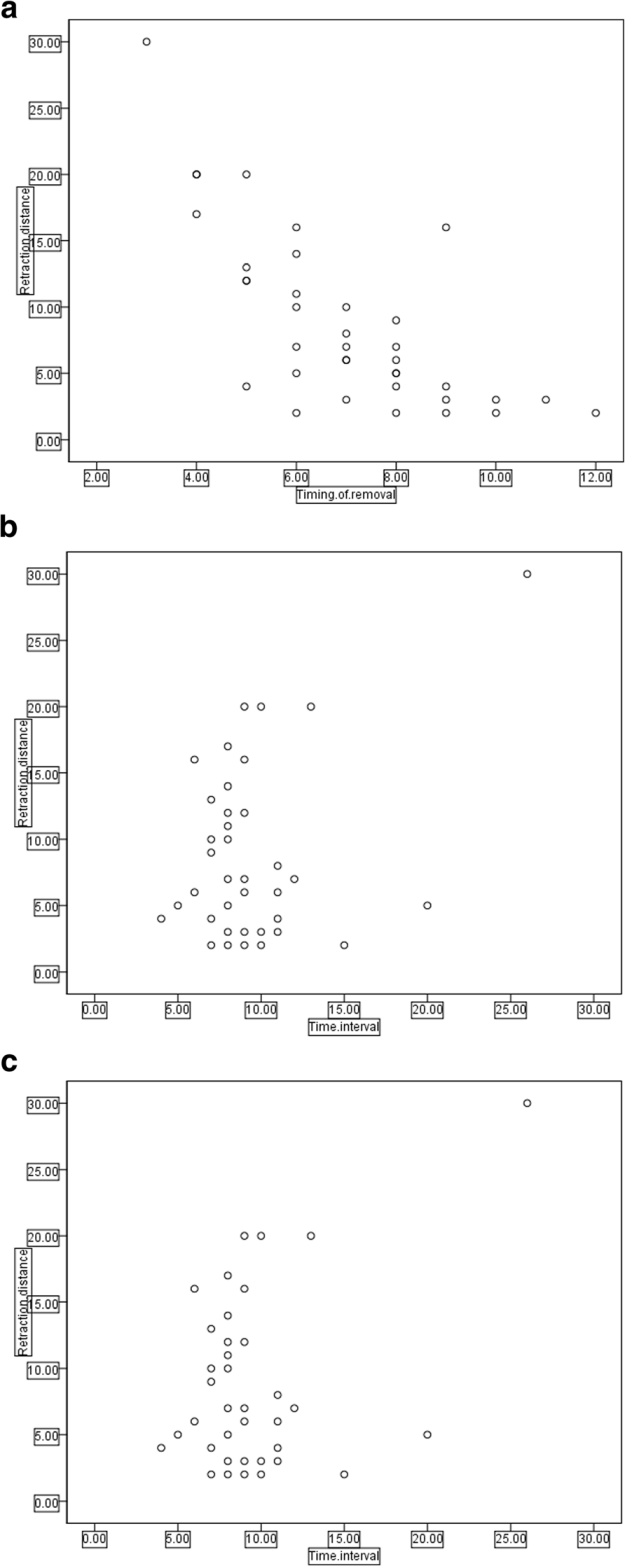
Scatter diagrams of the timing of removal(**a**), transport distance(**b**) and time interval of removal(**c**)

**Table 2:** Coefficients^a^

Model		Unstandardized Coefficients		Standardized Coefficients	t	Sig.
		B	Std. Error	Beta		
	(Constant)	27.070	3.960		6.836	.000
	Timing of removal	−2.808	.276	−.851	−10.170	.000
	Time interval	0.449	.140	.277	3.193	.003
	Distraction distance	−0.031	.044	−.066	−.717	.479

^a^ Dependent Variable: retraction distance

**Fig. 2:**
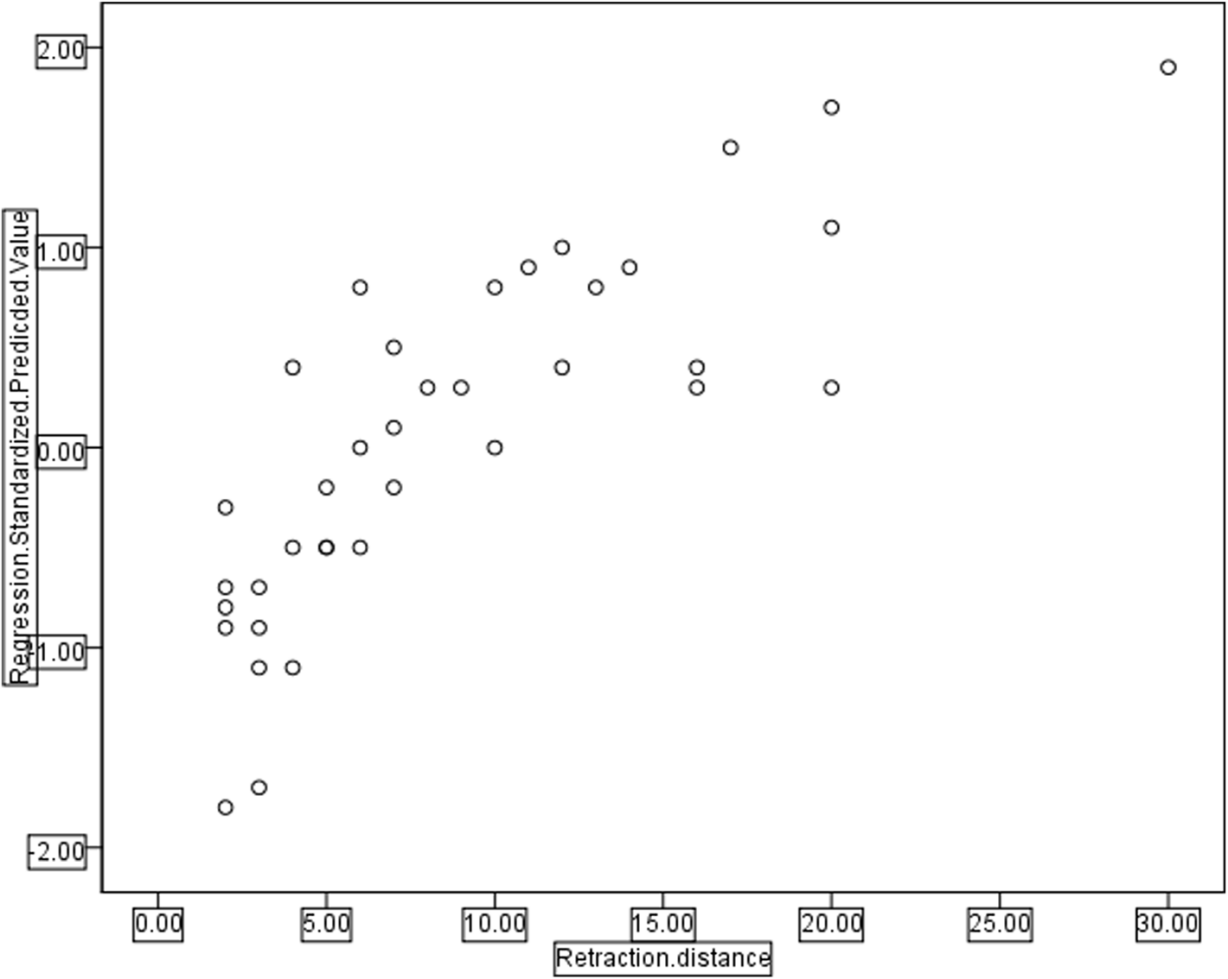
Scatter diagram of the regression model

**Fig. 3:**
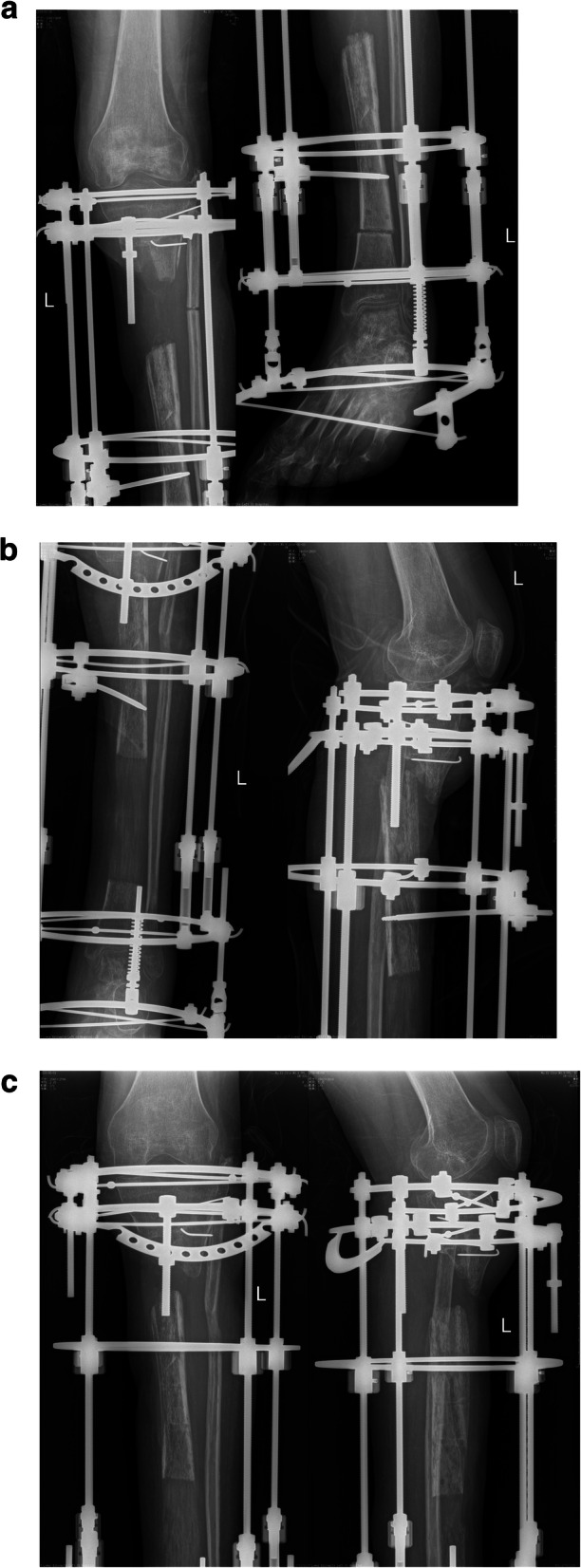
Patient with fracture and defect of tibia and fibula was treated by bone transport (**a**,**b**), in whom the X-Ray showed the TBS retracted 3.0 cm in a timing of removal of 3.5 months and a time interval of 25 days (**c**)

**Fig. 4:**
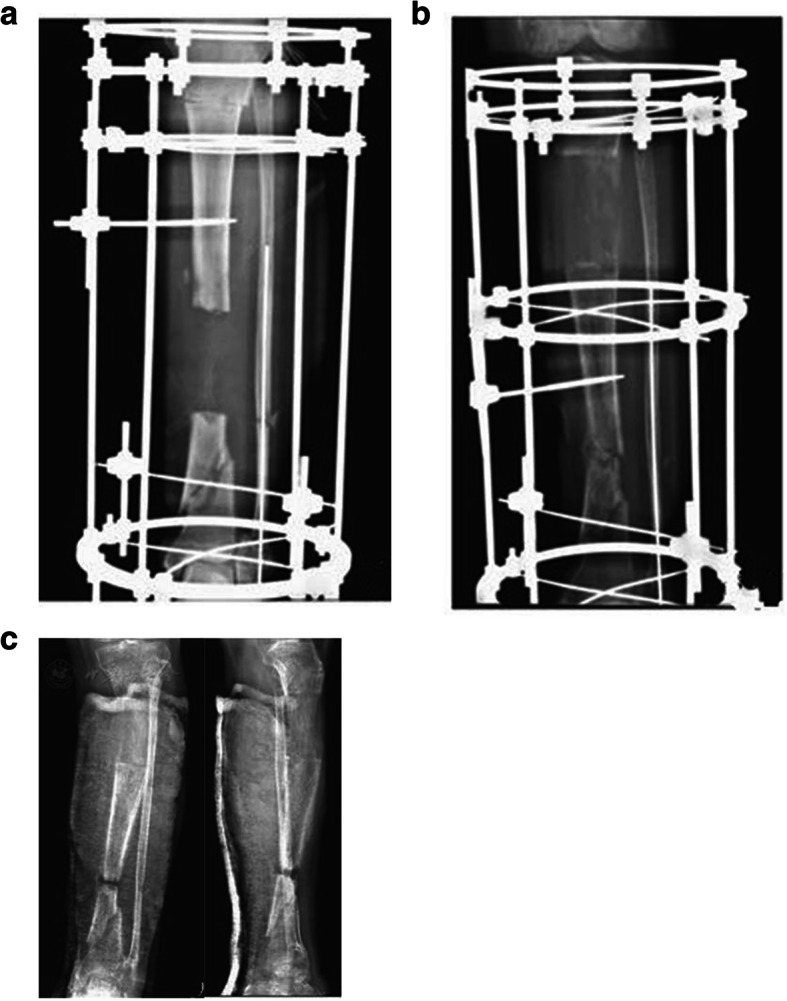
Patient with fracture and defect of tibia and fibula treated with bone transport (**a**,**b**), in whom the X-Ray showed the TBS retracted 4.0 mm in a timing of removal of 7 months and a time interval of 10 days (**c**)

## Discussion

Distraction osteogenesis is used to perform slow bone transport or lengthening using external distraction system or intramedullary distraction system after osteotomy. The resistance force (traction force) of the TBS suffered comes from two aspects during bone transport: one is generated from the distraction of the soft tissues around TBS, and the other is generated from the distracted callus at the lengthening site.

Although the periosteal connection was cut off after osteotomy, TBS still has adherent structure such as fascia, tendon or muscle, nerve, vessels, skin, tendons, ligaments and the connections among them. The magnitude of the traction force from soft tissue reported differs by different authors [10–13], which was mainly related to the transport distance, site and size of TBS. In general, the thicker the skeleton, or the longer the TBS and transport distance, the greater the force [10–13]. Horas et al. [4] used eight cadaveric thigh specimens to make a 60 mm bone defect at the middle femur, and then assessed the traction force required for 40-mm and 60-mm long of TBS using a novel type of intramedullary distraction system. The results showed that the traction force generated by soft tissue was linearly correlated with the transport distance. The force increased sharply at 0–10 mm transport distance, then slowly increased at 10–50 mm distance, sharply increased again to the maximum of 444.5 N at 50–60 mm transport distance. The traction force required for 60-mm long of TBS was higher than that for 40-mm long of TBS. The study indicated that transport distance and the size of TBS were related to the magnitude of traction force generated by its adjacent soft tissues. However, there was no report on the effect of the timing of removal of external fixator on the retraction distance.

The whole distraction osteogenesis process is divided into three phases: 1–2 weeks of latency period, then about 3–4 months of distraction period, and at last another 3–4 months of consolidation period [12, 15, 16]. The distraction callus gradually appears at distraction period, then gradually becomes dense, and finally matures at consolidation period. Full consolidation (maturation of consolidation) of the distracted callus can prevent the retraction of TBS.

There were still different opinions on which is the main force and which is the force causing retraction of TBS [10–12]. Aronson et al. [11] concluded that with the increase of transport distance, the traction force generated by distraction callus gradually increases, which is greater than that generated by soft tissues. However, Wolfson et al. [12] considered that soft tissue plays a decisive role in the generation of traction force. We believe that two kinds of traction forces of TBS endured change dynamically during bone transport and have different properties. In the early stage (within 3 months after bone transport), the traction force from the soft tissues is greater than that from the distraction callus and becomes an important role; in the middle stage (3–6 months after bone transport), the former reaches its peak and the latter gradually increases; in the late stage (> 6 months after bone transport), the former gradually decreases while the latter gradually reaches its peak and becomes an important role. The traction forces of TBS endured from the soft tissues and the unconsolidated distracted callus have elastic properties (as the tissue in the unconsolidated distracted callus is collagen aligned in linear bundles that can shorten like a spring until calcified) [10–14], which can make retraction of TBS. However, the traction force from the consolidated distracted callus has anti-retraction property and can prevent the retraction of TBS. Hu JZ et al. [7] reported an improved bone transport through conversion of external fixation to internal fixation after closure of the docking site, certain degree of retraction was observed in their study. In our study, the timing of removal in most patients was within 8 months after operation, only in 3 patients the timing of removal was more than 10 months after operation due to delayed consolidation, the distracted callus in all patients with retraction of TBS was not fully consolidated. The earlier the timing of removal, the larger the elastic traction forces, so the greater the retraction distance, while the later the timing of removal, the smaller the elastic traction forces, so the lesser the retraction distance, that is, the timing of removal is closely related to the elastic forces or the retraction distance of TBS.

Beside the traction force, the time interval is another important factor influencing the retraction distance of TBS. The longer the time interval, the more the retraction. In the typical case 1 of this study, the timing of TBS removal was earlier (3.5 months), the time interval was longer (25 days), which resulted in large retraction distance (30 mm). Our study showed that the timing of removal and time interval are the main factors affecting the retraction distance, especially the timing of removal had the greatest impact, followed by the time interval, but the transport distance and size of TBS are not the main factors.

Understanding the force and retraction phenomenon of TBS during bone transport is helpful to take corresponding measures to avoid adverse effect or complications. For example, the defect ends should be pressurized for 2 ~ 3 weeks when the docking site is closed in traditional bone transport [17]; In the case of early removal, another external fixation or quickly converted to internal fixation should be performed to avoid the adverse effect of more retraction on the healing of the docking site. Otherwise, more bone grafts are needed because it is difficult to complete the reduction of the retraction.

This study explored the causes and relevant factors of the retraction of TBS during Ilizarov bone transport. Those findings are helpful to understand the retraction of TBS, improve prognosis and reduce complications of bone transport in the treatment of bone defect.

## Conclusions

The traction forces of TBS endured from the soft tissues and the unconsolidated distracted callus have elastic properties and can make a retraction of TBS. The timing of removal and time interval are the main risk factors of the retraction of TBS. In the case of earlier removal, another external fixation or quick conversion to internal fixation should be performed to avoid the adverse effect of more retraction.

## Data Availability

Not applicable.

## Abbreviation

TBS: Transporting bone segment
